# Supplementary material for: Purposeful listening in challenging conditions: A study of prediction during consecutive interpreting in noise
Source: PLoS One. 2023 Jul 20;18(7):e0288960. doi: 10.1371/journal.pone.0288960 (PMC10359016; doi:10.1371/journal.pone.0288960)
Supplement: S1 Appendix — (DOCX) [file pone.0288960.s001.docx]

Appendix: Experimental items

| **Item** | **Sentence (Cloze rating % for the underlined word)** | Object name (Naming agreement %) |
| --- | --- | --- |
| 1 | In order to have a closer look, the dentist asked the man to open his mouth a little wider. (100) | mouth (100) |
|  |  | mouse (91.7) |
|  |  | bone (100) |
| 2 | In an emergency, we cannot use a lift; instead, we need to use the stairs for our safety. (95) | stairs (100) |
|  |  | stapler (83.3) |
|  |  | calculator (100) |
| 3 | If the sun comes out during a heavy shower, you can sometimes see a rainbow in the sky. (95) | rainbow (100) |
|  |  | radio (100) |
|  |  | goat (100) |
| 4 | The tourists expected rain when the sun went behind the cloud, but the weather got better later. (95) | cloud (91.7) |
|  |  | clown (100) |
|  |  | train (100) |
| 5 | The man didn't know the time because he forgot to wear the watch that he usually wears. (95) | watch (100) |
|  |  | washing machine (75.0) |
|  |  | tray (16.7) |
| 6 | Bob proposed and gave her a ring that had cost him half his monthly wage. (85) | ring (100) |
|  |  | ribbon (58.3) |
|  |  | letter (83.3) |
| 7 | The man was gathering honey, when he was stung by a bee and gave a cry. (90) | bee (100) |
|  |  | beard (100) |
|  |  | helmet (83.3) |
| 8 | People can easily go to the island on foot since the government built a bridge last year. (85) | bridge (91.7) |
|  |  | brick (83.3) |
|  |  | meat (50.0) |

| 9 | The traveller went to the desert because he wanted to ride a camel and go exploring. (95) | camel (83.3) |
| --- | --- | --- |
|  |  | camera (100) |
|  |  | barrel (33.3) |
| 10 | The woman found the room was too hot and humid, so to get some fresh air, she opened the window completely. (100) | window (91.7) |
|  |  | windmill (83.3) |
|  |  | globe (100) |
| 11 | The bird cannot fly because it injured its wing when it had a fight with another bird. (95) | wing (75) |
|  |  | witch (100) |
|  |  | flag (83.3) |
| 12 | Amber went to the dealership to purchase a new car the very next day. (50) | car (100) |
|  |  | castle (100) |
|  |  | bear (100) |
| 13 | To protect against an enemy's bullet or arrows, soldiers used to carry a shield all the time. (45) | shield (83.3) |
|  |  | sheep (100) |
|  |  | onion (83.3) |
| 14 | Before he began to draw, he sharpened his pencil and got out some paper. (100) | pencil (100) |
|  |  | penguin (100) |
|  |  | cherry (100) |
| 15 | In order to study, Karen sat down at her desk and opened her book. (80) | desk (100) |
|  |  | dentist (8.3) |
|  |  | chick (83.3) |
| 16 | In the night sky it is easier to see all the stars and the moon. (100) | star (91.7) |
|  |  | stamp (66.7) |
|  |  | key (100) |
| 17 | The maid dusted the books on the shelf every week. (55) | shelf (16.7) |
|  |  | shell (41.7) |
|  |  | pig (100) |

| 18 | The student went to the library to read a book but in the end he ended up chatting with his friends. (100) | book (100) |
| --- | --- | --- |
|  |  | bull (83.3) |
|  |  | dice (58.3) |
| 19 | Joan fed her baby some warm milk and then put him to bed. (70) | milk (91.7) |
|  |  | mirror (100) |
|  |  | salt (100) |
| 20 | She went to the beauty parlour to perm her hair in preparation for the party. (50) | hair (83.3) |
|  |  | helicopter (100) |
|  |  | bamboo (91.7) |
| 21 | One day, the caterpillar will turn into a beautiful butterfly and fly away. (90) | butterfly (100) |
|  |  | button (100) |
|  |  | giraffe (100) |
| 22 | Catherine carried her computer in a shoulder bag until she found it was giving her back problems. (100) | bag (91.7) |
|  |  | bat (75.0) |
|  |  | kiwi (91.7) |
| 23 | After every meal it’s good to brush your teeth or else chew gum. (100) | teeth (100) |
|  |  | teapot (75.0) |
|  |  | horse (100) |
| 24 | Dad carved the turkey with a knife for Christmas dinner. (95) | knife (100) |
|  |  | knight (83.3) |
|  |  | rabbit (83.3) |
| 25 | He loosened the tie around his neck and immediately felt better. (95) | neck (91.7) |
|  |  | nest (83.3) |
|  |  | handcuff (91.7) |
| 26 | A flat tyre forced Katy to pull up at the side of the road and call for assistance. (75) | road (100) |
|  |  | rope (100) |
|  |  | glasses (75.0) |

| 27 | The referee blew his whistle to signal the end of the match. (70) | whistle (66.7) |
| --- | --- | --- |
|  |  | whisk (25.0) |
|  |  | pen (75.0) |
| 28 | To make sushi, the chef went to the market to buy some fish early in the morning. (70) | fish (n/a) |
|  |  | finger (n/a) |
|  |  | scarf (100) |
| 29 | John was very tired so he decided to go straight to bed and sleep. (90) | bed (100) |
|  |  | belt (83.3) |
|  |  | glove (91.7) |
| 30 | To reach the roof, the workman climbed up the ladder that was against the wall. (65) | ladder (83.3) |
|  |  | laptop (66.7) |
|  |  | bench (58.3) |
|  | | |
